# Perceived work-related stigma in healthcare workers and presence of post-traumatic stress disorders (PTSD) symptoms

**DOI:** 10.1192/j.eurpsy.2025.1968

**Published:** 2025-08-26

**Authors:** C. Vincent, H. Scarlett, M. Melchior, C. Vuillermoz

**Affiliations:** 1 Team of social epidemiology, Institut Pierre Louis d’Epidémiologie et de Santé Publique; 2 Inserm; 3 Institut Pierre Louis d’Epidémiologie et de Santé Publique, Paris; 4 Université de Versailles, Saint-Quentin-en-Yvelines, France

## Abstract

**Introduction:**

Previous research has demonstrated a relationship between work-related stigma during the COVID-19 pandemic and posttraumatic stress disorder (PTSD). Whilst it is possible that shared social characteristics explain the link between stigma and PTSD, this article hypothesizes that work-related stigma is itself associated with symptoms of PTSD.

**Objectives:**

This study examined whether work-related stigma was associated with PTSD symptoms one year after the onset of the COVID-19 pandemic in a sample of French healthcare workers (HCWs).

**Methods:**

Data were based on an online survey that recruited HCWs working during the first wave of the COVID-19 pandemic. The presence of PTSD symptoms was assessed using the Posttraumatic Stress Disorder Checklist (PCL-5) with a cut-off score of ≥33. Work-related stigma was defined as the perception of having been treated differently and unfairly because of one’s profession.The relationship between work-related stigma and PTSD symptoms was tested using propensity scores including all relevant confounding factors: financial and social support, and work-related,or pandemic-related factors.

**Results:**

Among the 655 respondents, 71.6% were physicians, 11.1% were nurses, and 17.3% had other occupations. A total of 53 participants (8.7%) presented symptoms of PTSD. We found an association between work-related stigma and PTSD symptoms (OR 2.13, 95% CI [1.04;4.35]) one year after the onset of the COVID-19 pandemic adjusted for propensity scores including relevant confounding factors.

**Conclusions:**

Our findings suggest that work-related stigma is associated with PTSD symptoms among HCWs. This association was independent of social characteristics known to influence both stigma and PTSD. Work-related stigma may be a propitious occupational risk factor to target in order to improve HCWs work conditions and mental health.

**Disclosure of Interest:**

None Declared

